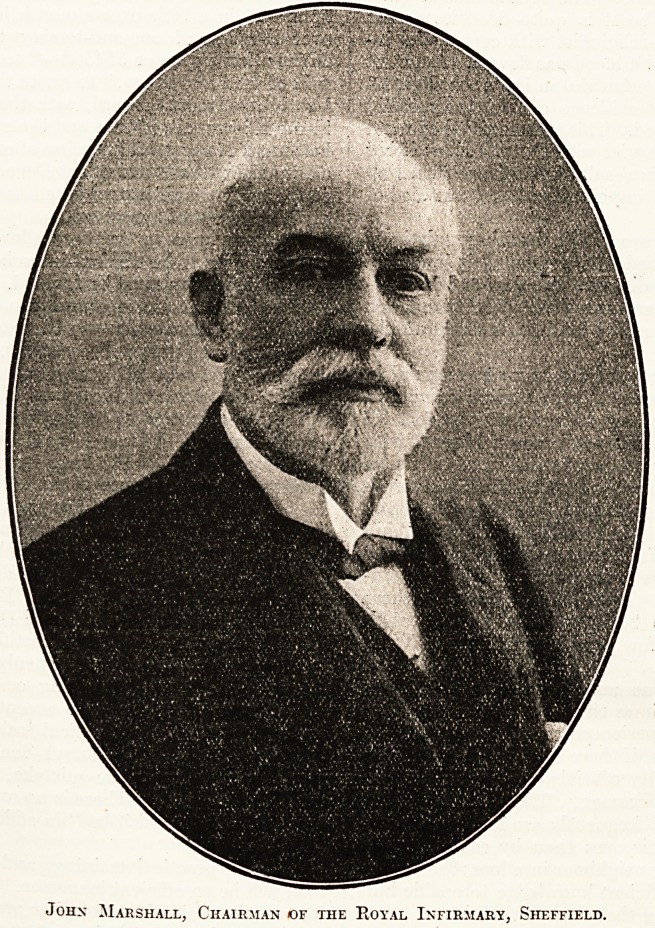# John Marshall, Chairman of the Royal Infirmary, Sheffield

**Published:** 1912-04-27

**Authors:** 


### EMINENT CHAIRMEN SERIES.

### JOHN MARSHALL, Chairman of the Royal Infirmary, Sheffield.

### AN APPRECIATION BY A COLLEAGUE.

That the charitable institutions of this country express in no small way one of the national characteristics few can deny, and that such charities are conducted ably by a system of boards of management, with a chairman elected from amongst them, is a well recognised fact.

It is the pleasant task of the writer to try and express the warmth of feeling in which the object of this article is held both by his colleagues on the board and, in no less a degree, by those on the honorary medical staff.

Mr. Marshall joined the board of the Royal Infirmary in 1875, and from the outset showed himself a most hard - working and enthusiastic member, and on the retirement of the chairman, the late Mr. Brooksbank, he was unanimously appointed to the vacant chair in 1890.

John Marshall, Chairman of the Royal Infirmary, Sheffield.

He has held and continues to hold this post with distinction and with ever-increasing benefit to the institution. Mr Marshall has not been content to pay sole attention to the financial side of the house, but has thrown himself, heart and soul, into the scientific side, and as a result of this the Infirmary is thoroughly and efficiently equipped with all the new and recent developments which modern scientific treatment demands.

The keynote of Mr. Marshall's eminent success as chairman is to be found in the delightful harmony which exists between the board of management and the honorary medical staff, and I venture to doubt if there is any institution in the country where such universally cordial relations exist. As a means of conveying to our highly esteemed chairman our warmth of good feeling, and as a slight token of recognition of his invaluable services to the institution, he was presented a few years ago with a handsome silver dessert centre-piece by all his colleagues on the board and medical staff. Many of the younger generation of resident medical officers owe him a deep sense of gratitude for the keen interest he has taken in them, both during their term of office and subsequently, where in many instances he has been instrumental in helping them to attain posts on the honorary medical staff, not only at the Infirmary, but also at similar institutions. He has always shown the keenest interest in the "young doctor," and has ever made it his labour of love to further his interests.

The enormous amount of work and responsibility which Mr. Marshall has shared with his colleagues since 1890 can be judged by the rapid growth of buildings — a house for nurses, an ophthalmic block of 40 odd beds with an exceedingly good operation-theatre, and a very large general operation-theatre, with suite of rooms and an excellent ventilating apparatus, completed in 1899, and since then, owing to the enormous increase in demand for beds, a completely self-contained modern surgical block of 180 beds accommodation, with two operation-theatres with necessary suites of rooms. This latter building has been in use just over a year.

## Figures and Tables

**Figure f1:**